# Early Nodal Metastasis in an 81-Year-Old Woman With Subcentimeter Retroareolar Invasive Ductal Carcinoma: A Case That Defies Indolence Expectations

**DOI:** 10.7759/cureus.105719

**Published:** 2026-03-23

**Authors:** Christopher M Ahmad, Rachana Tadakamalla, Rae-Anne Kastle, Andrea Weitoschova, Amer Abboud

**Affiliations:** 1 Internal Medicine, Kansas City University, Joplin, USA; 2 Pathology, Kansas City University, Joplin, USA; 3 General Surgery, Kansas City University, Joplin, USA; 4 Obstetrics and Gynecology, Kansas City University, Joplin, USA; 5 Pathology and Laboratory Medicine, Humboldt Park Health, Chicago, USA

**Keywords:** axillary lymph node metastasis, breast cancer, core needle biopsy, elderly patient, geriatric oncology, invasive ductal carcinoma, luminal a subtype

## Abstract

Retroareolar invasive ductal carcinoma (IDC) represents an anatomically distinct subset of breast cancers that may evade early clinical detection. In elderly patients, small, estrogen receptor (ER)-positive tumors with low proliferative indices are often presumed to follow an indolent course. We report the case of an 81-year-old woman diagnosed with a 0.6-cm Grade II/III retroareolar IDC exhibiting strong ER expression, low Ki-67 (~6%), and human epidermal growth factor receptor 2 (HER2) negativity, yet with synchronous axillary lymph node metastasis confirmed at initial biopsy. This case underscores the limitations of relying on tumor size, age, and proliferation markers alone to estimate metastatic risk and highlights the importance of comprehensive axillary evaluation, even in clinically and biologically favorable presentations.

## Introduction

Breast cancer remains the most frequently diagnosed malignancy among women; it is estimated that 2.3 million new cases are diagnosed each year globally, with invasive ductal carcinoma (IDC) accounting for the majority of cases [1,2]. In older adults, breast tumors are often assumed to exhibit less aggressive behavior [3], particularly when characterized by small size, hormone receptor positivity, and low proliferative indices [4,5]. These assumptions frequently influence decisions regarding staging intensity and therapeutic aggressiveness.

Lesions are deemed retroareolar if they are located within two cm of the nipple-areolar complex (NAC) [6]. Tumors arising in the retroareolar or central breast region represent a relatively uncommon anatomic subset and may pose diagnostic challenges due to their location beneath the NAC. Lesions involving or located near the NAC can be difficult to fully characterize with conventional imaging alone, and advanced imaging modalities, such as breast magnetic resonance imaging, have increasingly been used to evaluate occult NAC involvement and assess tumor proximity to the nipple [6,7]. In addition, masses located directly beneath the nipple may be partially obscured on imaging, potentially contributing to missed retroareolar tumors on routine screening examinations [8]. As a result, some tumors in this region may be detected only after nodal metastasis has occurred or at the time of diagnostic evaluation of the primary lesion.

We describe a case of subcentimeter retroareolar IDC in an elderly patient with otherwise favorable biological features, in whom axillary lymph node metastasis was identified at the time of diagnosis. This case highlights the importance of maintaining vigilance during staging, regardless of patient age or presumed tumor indolence.

## Case presentation

An 81-year-old woman underwent routine breast imaging that identified a lesion within the retroareolar region of the left breast. She denied nipple discharge, skin changes, or nipple retraction. Clinical breast examination did not reveal a palpable mass, and no axillary adenopathy was appreciated on physical examination. The patient was managed at Humboldt Park Health in Chicago, Illinois, USA.

**Figure 1:**
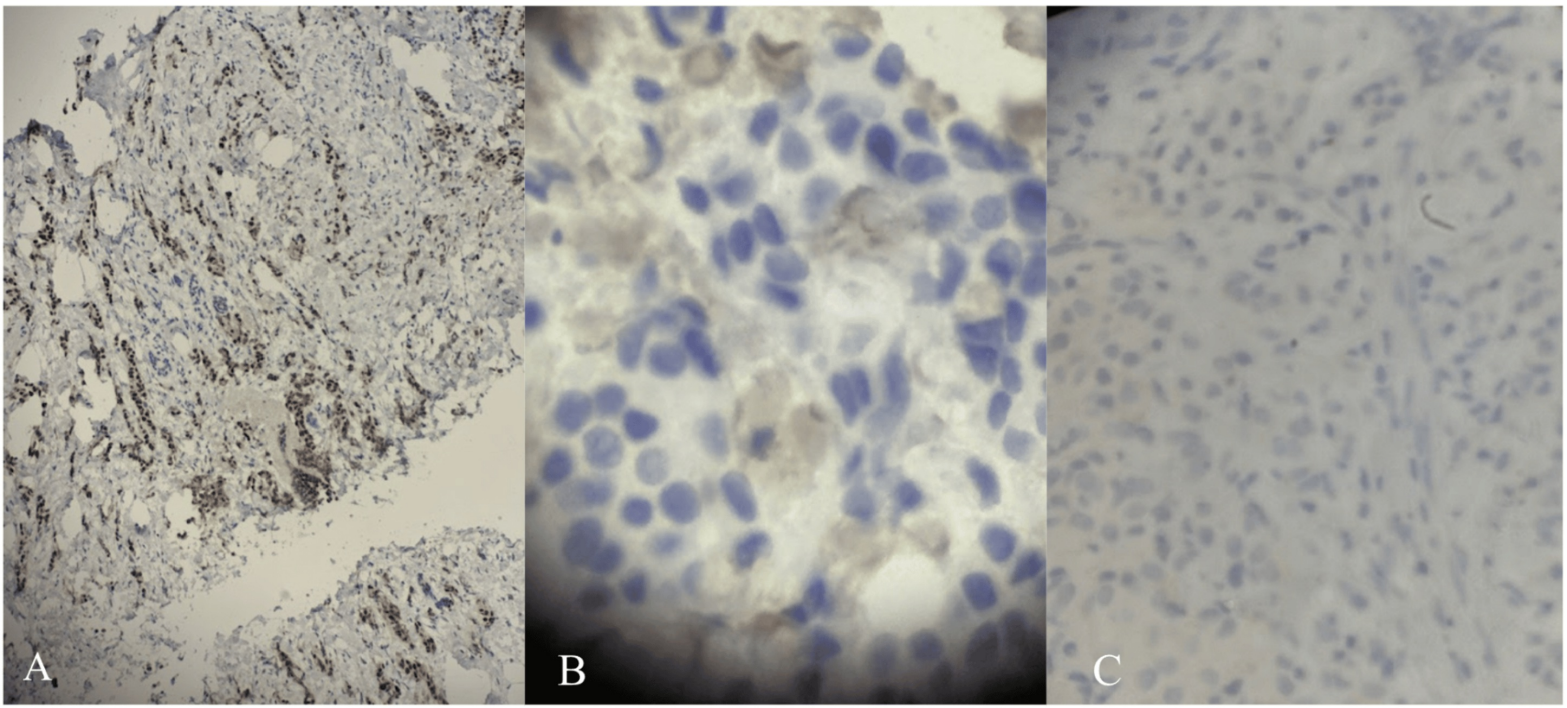
Immunohistochemical staining of the retroareolar invasive ductal carcinoma demonstrating receptor expression patterns. (A) Estrogen receptor (ER) staining showing strong, diffuse nuclear positivity in malignant epithelial cells (immunohistochemistry, ER stain; original magnification ×200). (B) Progesterone receptor (PR) staining demonstrating absence of nuclear staining in tumor cells (immunohistochemistry, PR stain; original magnification ×200). (C) Human epidermal growth factor receptor 2 (HER2) staining demonstrating absence of membranous staining consistent with HER2-negative status (score 0) according to ASCO/CAP criteria (immunohistochemistry, HER2 stain; original magnification ×200). ASCO: American Society of Clinical Oncology; CAP: College of American Pathologists; ER: estrogen receptor; PR: progesterone receptor; HER2: human epidermal growth factor receptor 2.

Ultrasound-guided core needle biopsy of the retroareolar lesion was performed. Four cores were obtained; three demonstrated IDC, Grade II/III. The largest contiguous focus of invasive carcinoma measured 0.6 cm, corresponding to clinical stage cT1b disease. Immunohistochemical analysis, shown in Figure 1, revealed estrogen receptor (ER) positivity in approximately 90% of tumor cells, progesterone receptor (PR) negativity, and human epidermal growth factor receptor 2 (HER2) negativity (score 0). The Ki-67 proliferation index was estimated at approximately 6%. E-cadherin and CKAE1/AE3 staining supported ductal differentiation. These clinicopathologic findings are summarized in Table 1.

**Table 1:** Clinicopathologic and immunohistochemical profile of the primary retroareolar breast carcinoma and associated axillary metastasis. HER2: human epidermal growth factor receptor 2; AJCC: American Joint Committee on Cancer; ER: estrogen receptor; PR: progesterone receptor.

Feature	Description
Age	81 years
Tumor size	0.6 cm (cT1b)
Histologic grade	Grade II/III
Estrogen receptor (ER)	Positive (90%)
Progesterone receptor (PR)	Negative
HER2/neu status	Negative (score 0)
Ki-67 index	6%
Nodal status	Positive (metastatic ductal carcinoma)
Clinical stage	cT1bN1M0 (AJCC 8th edition)
Pathologic stage	pT1bN1M0 (AJCC 8th edition)

**Figure 2:**
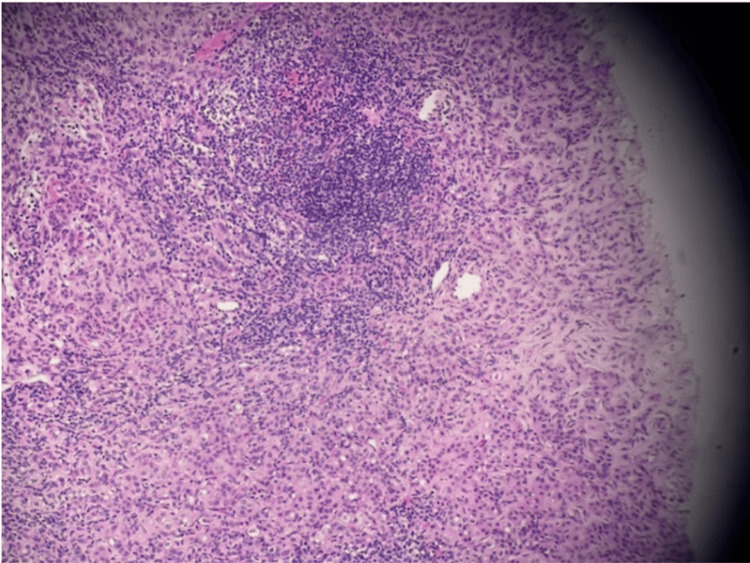
Axillary lymph node metastasis. Core needle biopsy of the axillary lymph node demonstrating metastatic ductal carcinoma replacing native nodal architecture (hematoxylin and eosin stain; original magnification ×100).

During the same diagnostic session, imaging identified an abnormal left axillary lymph node (Figure 2). Ultrasound-guided core biopsy of this node was performed, and histopathologic evaluation confirmed metastatic ductal carcinoma, establishing nodal involvement consistent with N1 disease. Immunohistochemical staining for E-cadherin demonstrated strong membranous expression in metastatic tumor cells, supporting ductal differentiation and confirming the diagnosis of metastatic IDC.

**Figure 3:**
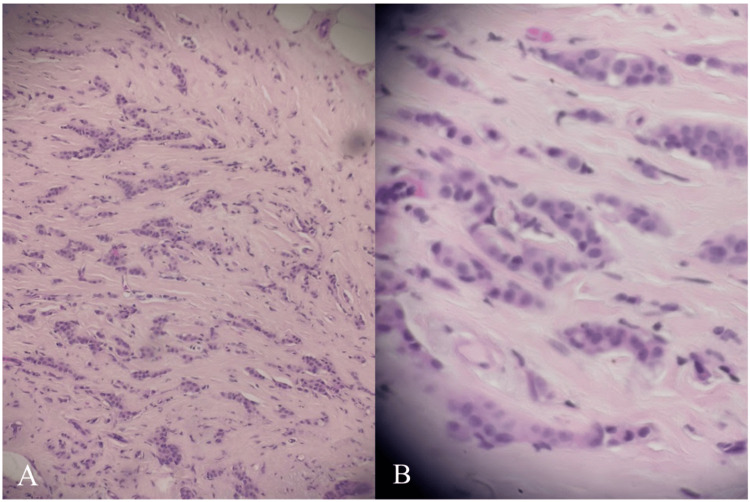
Histopathology of retroareolar invasive ductal carcinoma. (A) Hematoxylin and eosin-stained section showing infiltrative malignant epithelial cells arranged in irregular cords and nests within a desmoplastic stroma (H&E stain; original magnification ×100). (B) Higher-power view demonstrating tumor cell nuclear atypia and stromal invasion characteristic of IDC (H&E stain; original magnification ×400). H&E: hematoxylin and eosin; IDC: invasive ductal carcinoma.

Figure 3 depicts the histopathologic evaluation of the retroareolar lesion, demonstrating infiltrative malignant epithelial cells arranged in irregular cords and nests within a desmoplastic stroma, consistent with IDC.

Gross examination of the breast biopsy specimen demonstrated yellow-pink to orange-tan tissue fragments measuring 6 × 0.2 × 0.2 cm in aggregate, with the longest individual core measuring 3 × 0.2 cm. The lymph node biopsy specimen consisted of pink-to-orange-tan cores measuring 5 × 0.1 × 0.1 cm in aggregate, with the longest core measuring 2.5 × 0.1 × 0.1 cm. Cold ischemia time for both specimens was approximately five minutes, and fixation in 10% neutral buffered formalin exceeded six hours but was less than 72 hours. These findings established the diagnosis of a subcentimeter retroareolar IDC with synchronous axillary lymph node metastasis.

Based on the presence of a 0.6-cm IDC with biopsy-confirmed axillary lymph node metastasis and no evidence of distant disease on initial evaluation, the tumor was assigned a clinical stage of cT1bN1M0 according to the American Joint Committee on Cancer (AJCC) 8th edition staging system, corresponding to stage IIA breast cancer. Following histopathologic confirmation of IDC measuring 0.6 cm with metastatic involvement of the sampled axillary lymph node, the tumor met criteria for pathologic stage pT1bN1M0 according to the AJCC 8th edition staging system. Following confirmation of IDC with axillary lymph node metastasis, the patient was referred for multidisciplinary oncologic evaluation, including consultation with surgical oncology and medical oncology for definitive management. Management planning included consideration of definitive surgical treatment with axillary staging and endocrine therapy, given the tumor’s strong ER positivity. Due to limitations in the clinical record available for this report, detailed documentation regarding definitive surgical management and adjuvant therapy was not available at the time of manuscript preparation. The present report, therefore, focuses on the patient’s diagnostic presentation and pathologic findings at the time of initial staging.

## Discussion

In elderly patients, small, hormone receptor-positive breast cancers are often presumed to carry a low risk of nodal involvement, particularly when proliferation indices such as Ki-67 are low [4,5]. This assumption can influence decisions regarding axillary evaluation and overall staging. However, the present case illustrates a clear exception to this paradigm.

Despite advanced patient age, small tumor size, strong ER expression, and a low Ki-67 index, axillary metastasis was present at the time of diagnosis. This finding reinforces the notion that tumor size alone does not preclude early dissemination, as metastatic competence may arise early during tumor evolution, even when the primary lesion remains small [9]. The retroareolar location may have contributed to delayed detection. Central breast tumors can lack early palpable findings or overt nipple changes; however, clinical examination has been reported to be more sensitive than routine mammography for detecting lesions in the retroareolar region [6,7]. Additionally, lymphatic drainage patterns of the subareolar plexus may facilitate early nodal involvement, even in small primary tumors [6].

The tumor’s ER-positive, PR-negative, and HER2-negative profile is also clinically relevant. While ER-positive tumors in elderly patients are often managed with endocrine therapy alone, PR loss in ER-positive tumors has been associated with less favorable outcomes and altered endocrine responsiveness compared with ER-positive/PR-positive tumors [10]. Some studies suggest that ER-positive/PR-negative cancers behave more aggressively than their PR-positive counterparts and may demonstrate higher recurrence risk or altered endocrine responsiveness despite otherwise “luminal” features [10].

In this context, nodal involvement substantially alters staging and therapeutic decision-making in breast cancer, as axillary lymph node metastasis remains a major determinant of patient prognosis and survival outcomes [11]. This case underscores the limitations of relying on Ki-67 as a solitary marker of aggressiveness. Ki-67 can be affected by sampling variability, differences in scoring methodology, and the lack of universally consistent cutoffs across laboratories, which complicate its use as a sole predictor of metastatic potential [12]. A low Ki-67 index should, therefore, be interpreted as one component of an integrated assessment rather than as reassurance that lymphatic dissemination is unlikely, particularly in anatomically central tumors and in cases with PR-negative status [13]. Low proliferation indices do not exclude the possibility of metastatic spread, particularly when anatomical factors and receptor profiles intersect [9].

Although tumor size is a major component of breast cancer staging, small IDCs are not uniformly associated with negligible metastatic risk [14]. Nodal involvement has been documented even in lesions under 1 cm, and the probability of axillary spread appears to vary based on histologic grade, receptor profile, and tumor microenvironment rather than size alone [15]. This case reinforces that subcentimeter tumor burden does not guarantee biologic containment and supports a staging approach that remains attentive to features beyond tumor diameter [16].

The retroareolar location may contribute to both diagnostic complexity and metastatic behavior. Central breast tumors can be clinically subtle, sometimes lacking early palpable findings or visible nipple changes, which may delay recognition even when routine imaging is performed [6,7]. In addition, the subareolar region is characterized by a rich lymphatic network that communicates efficiently with the axillary basins, providing a plausible anatomic mechanism for early nodal involvement even when the primary lesion is small [17]. Viewed through this lens, the discordance between the tumor’s limited size and its synchronous nodal metastasis becomes more biologically coherent [11]. Taken together, these considerations support comprehensive axillary evaluation even when clinical and biologic features initially appear favorable.

Beyond conventional clinicopathologic parameters, this case raises the possibility of intratumoral heterogeneity that may not be fully captured by core biopsy sampling. Even small IDCs may contain subclonal populations with enhanced metastatic potential that are not proportionally reflected in the sampled tissue. Core needle biopsy provides a limited representation of tumor architecture and biology, and low proliferative indices measured from a restricted tumor area may underestimate aggressive cellular subsets within the lesion [18]. The presence of synchronous nodal metastasis in this case suggests that metastatic competence may arise early in tumor evolution, potentially independent of bulk tumor proliferation. This highlights the limitation of relying exclusively on sampled histologic features to estimate systemic risk.

Additionally, this case underscores the importance of integrating imaging findings with pathologic interpretation at the time of diagnosis. The abnormal axillary lymph node was identified during the same diagnostic encounter as the primary lesion, allowing for immediate tissue confirmation. This coordinated diagnostic approach prevented under-staging and ensured accurate pathologic classification from the outset. In elderly patients, where there may be a tendency toward de-escalation of staging, maintaining vigilance when radiographic abnormalities are present remains critical [19]. The early recognition of nodal involvement in this patient substantially altered staging and likely informed subsequent management decisions. This reinforces that comprehensive initial evaluation, rather than assumptions based on age or tumor size, remains central to precision oncologic care. Management planning included consideration of definitive surgical treatment with axillary staging and endocrine therapy given the tumor’s strong ER positivity, with treatment decisions to be finalized following multidisciplinary oncology evaluation.

Limitations

This case report is limited to its single-patient design and absence of long-term follow-up data at the time of writing. As with all case reports, conclusions regarding causality or frequency cannot be inferred. Additionally, molecular profiling beyond standard immunohistochemistry was not performed, which may have further characterized underlying tumor heterogeneity. Furthermore, genomic profiling assays such as Oncotype DX or MammaPrint were not documented in the available clinical record, which may have provided further insight into the tumor’s genomic risk profile and biologic behavior. Nonetheless, the case highlights an important clinical exception that contributes to the understanding of metastatic risk in small, biologically favorable breast cancers. Additionally, detailed follow-up regarding definitive treatment and long-term clinical outcomes was not available at the time of manuscript preparation.

## Conclusions

This case illustrates that axillary lymph node metastasis can occur even in elderly patients with subcentimeter, ER-positive, low proliferative IDC. Tumor size and low Ki-67 index alone should not be interpreted as guarantees of biologic indolence. Retroareolar location and PR-negative status may contribute to early dissemination through both anatomic and biologic mechanisms. Comprehensive staging, including axillary evaluation when indicated, remains essential to accurate risk assessment. Individualized, biology-informed management, rather than reliance on age or tumor diameter alone, remains the best practice in geriatric breast oncology.
